# Optimal settings and advantages of drones as a tool for canopy arthropod collection

**DOI:** 10.1038/s41598-022-22446-z

**Published:** 2022-10-26

**Authors:** Jamie C. Madden, Émile Brisson-Curadeau, Jessica P. Gillung, David M. Bird, Kyle H. Elliott

**Affiliations:** 1 Department of Natural Resource Sciences, McGill University, Montreal, Canada; 2 Lyman Entomological Museum, Department of Natural Resource Sciences, McGill University, Montreal, Canada

**Keywords:** Ecology, Biodiversity, Conservation biology

## Abstract

The growing field of aeroecology is limited by difficulties associated with sampling in the air column. Aerial insects are particularly hard to sample, despite being the main prey in the air column, with some recent studies attempting to use drones as a collection method. We conducted a study to determine the optimal drone settings for collecting insects above the canopy, where drones are seldom used. By attaching a net to the body of a small, commercial drone, we tested yield from different height, speed, and net settings in wetlands, as well as compared insect diversity across different habitat canopies. Height was the most important setting; grazing the canopy yielded significantly more insects than flying one meter above it. Speed, drone type, and net size did not influence the number of insects caught per trial. Wetland canopies had higher abundance, diversity, and species richness in its arthropod populations compared to forest canopies or lakes. Compared to the yield of Lindgren funnels—a traditional sampling method in entomology—drones captured higher diversity and abundance of insects in a fraction of the time. This study confirms that drones are an efficient and accurate way to collect canopy arthropods.

## Introduction

The aerosphere has been studied extensively from a meteorology standpoint, but often ignored as an ecological domain^1^. For example, while biologists will typically label a bat’s habitat as ‘fields’ or ‘forests’, the connective habitat—the air—is often excluded^2^. The exclusion of this space is then extended into research and policy, where habitat conservation efforts have primarily focused on terrestrial and aquatic issues, all the while many threatened species rely on the aerial habitat^3^. To that end, habitat classification systems such as the International Union for Conservation of Nature (IUCN) disregard the aerial habitat completely by including only terrestrial and aquatic options^3^. It is therefore unsurprising that aerial habitats have been historically undefined and underrepresented, culminating in a lack of protection. In addition to the absence of conservation, and perhaps due to the complicated logistics of sampling in the air column, we know very little about the ecology of the airspace^2^. These challenges are now being addressed in the growing field of aeroecology, which studies how insects, birds, bats and other wildlife use the airspace^3^.

Aerial insects living just above habitat canopies are one such group that has been understudied in ecology due to logistical difficulties. Little is known about these populations that reside in and above forest canopies, as reaching them is challenging and often requires complicated canopy infrastructure or manned aircrafts^4^. Nonetheless, these insects are functionally important to the ecosystems where they are found. Canopy arthropods are involved in facultative mutualism with canopy plants as pollinators, act as both herbivores and predators in the food chain, and participate in decomposition, nutrient cycling, and energy transfer^5,6^. Insects found in the grassy canopies of wetlands have additional roles to their forest canopy counterparts, often serving as bioindicators of the health of the habitat^7,8^. Canopy-dwelling and aerial insects are also vital prey sources for aerial insectivores such as birds and bats, both of which are declining in North America^9^. However, the absence of data on trends in aerial insects means that it is unclear whether prey declines are a major cause of declines in aerial insectivores. With insect biomass globally shrinking, there is effort needed to find an efficient way to study and define these populations^10^.

Unoccupied Aerial Vehicles (UAVs or drones) have recently become prevalent in biology studies with the introduction of clearer regulations and diminished restrictions of the airspace^11,12^. In addition to legal simplicity, there are many reasons that drones are such convenient tools for ecologists. For example, while vehicle accidents are the most common cause of mortality for wildlife biologists, drones reduce risk associated with vehicles by allowing researchers to sample and take pictures completely remotely^13^. They also provide a much cheaper, more-environmentally friendly, and less time-consuming alternative to traditional aerial survey techniques such as light aircraft (e.g. helicopters and planes), canopy cranes and walkways.

A few recent studies have tested UAV potential for sampling the airspace for insect collection in entomology studies, as well as in other fields such as bat research^14–19^. The common technique used in these entomology UAV studies is to attach an insect sweep net to the body of a drone, with the insects in the air column being collected by the flying net. While the most exhaustive test of this method has focused on crop pests on agricultural fields^14^, no studies to date have explored the use of this technique over forest canopies. Additionally, none have examined the most effective and efficient drone settings to collect insects using a rotary drone, which has greater maneuverability than a fixed wing. In this study, we attempted to validate the use of drones and sweep nets as a method for canopy arthropod collection in multiple habitats and compare those results to those from conventional methods (Lindgren funnel). Furthermore, we tested differences in drone speed, height, and net diameter on abundance and diversity of arthropods collected.

Specifically, we wished to determine the optimal technique and settings for drone flying to collect insects and determine if drones and sweep nets are an efficient tool in canopy entomology. To that end, we developed a novel net design attached to a small, commercial drone that can be flown with a basic drone license. With the four factors being considered (height, speed, net diameter, and drone), we hypothesized that the highest insect yields will come from fast velocity flights, low height flights, and with a large net diameter, with the drone used having no effect. The second objective was to test our methodology on four different habitats in Quebec; coniferous forest canopies, deciduous forest canopies, grassy wetland canopies, and above bodies of water. Insect abundance and diversity was compared across habitats, as well as to traditional Lindgren funnels used for understory insect captures. Here we hypothesize that wetlands will have the greatest diversity and abundance of canopy arthropods, followed by deciduous forest canopies, lakes, and coniferous forests canopies.

## Methods

### Study site

All research was done within the Kenauk Nature Reserve in Montebello, Quebec, Canada. The reserve is a protected watershed and wildlife corridor, containing coniferous and deciduous forests, wetlands, and over 60 lakes.

### Optimal drone settings

**Figure 1:**
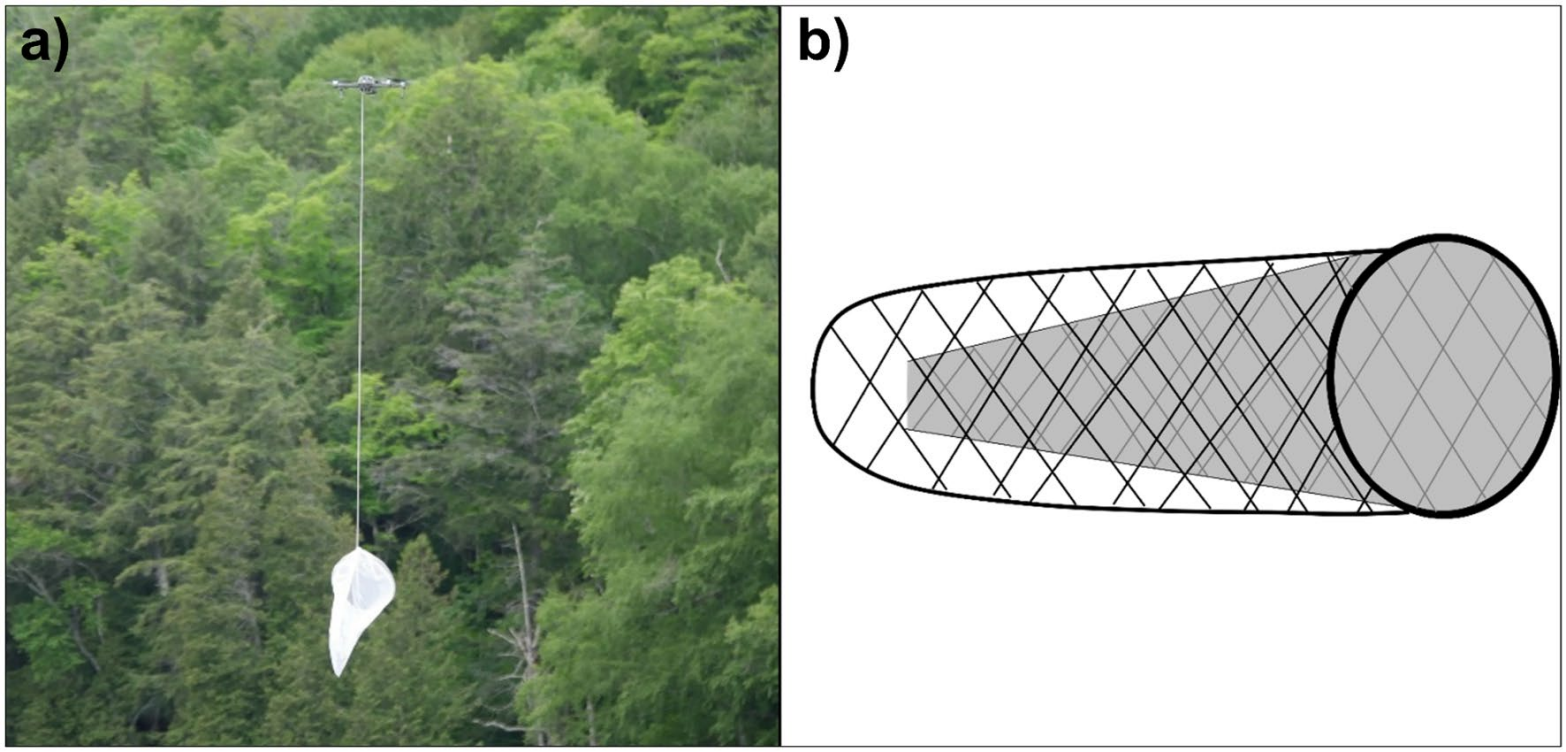
(**a**) Mavic 2 drone with net attached (**b**) illustration of our net design; solid grey represents the second conical layer of tulle netting.

Optimal techniques for flying a drone and a sweep net were studied at two wetland sites in September 2021. Two drones, the Mavic 2, and the Phantom IV, manufactured by DJI (Shenzhen, China), were used to facilitate pairwise comparisons. These were flown simultaneously for 24 flights over two days to compare different drone flying techniques. The settings compared were height above the canopy (0 m high vs. 1 m high), speed (10 km/hr. vs. 20 km/hr.), net diameter (12 inches or 305 mm vs. 18 inches or 457 mm), and drone type used (Mavic vs. Phantom). A ‘zero-meter’ height refers to the net grazing the canopy at vegetative sites or flying as close to the water at lake sites as possible, while a height of ‘one meter’ describes the net hanging approximately one meter above the foliage or water. We flew the drones simultaneously around the site at random for 4 min, after which the drone descended, a researcher shut the opening of the net with a piece of cardboard, and the drone was landed. The cardboard used to shut the net was rigged with a small flap, with which an arm could be reached inside and collect the insects without any others escaping. We then collected the insects into ethanol-filled tubes for laboratory identification. The nets used in all sections of this study were specially welded and sewed for this purpose. Tulle was chosen as the ideal material for the nets, as it is both very fine to capture all insect sizes, and stiff enough to stay open and not collapse into itself. The tulle was sewed onto welded frames in two layers, one outer layer which made up the net itself, and one inner conical layer which prevented insects from escaping once captured in the air (Fig. 1).

### Canopy diversity and abundance

To study the diversity between ecosystem sites, we selected three sites on the Kenauk Nature Reserve for each habitat type (coniferous forest, deciduous forest, wetland, and lake). The study design included all 12 sites being sampled four times over a 16-day period. The drone was unable to fly in rain or high winds, and was thus only flown on calm, clear days.

For this part of the study, a Mavic 2 drone was used. At each site, we would fly the drone and hanging net around randomly for three minutes, at zero-meter height (grazing the canopy), 10 km/hr, and with alternating net sizes. At the beginning of the study, a secondary drone was deployed to act as a “spotter” for the drone with the net, where the operator could advise on height adjustments to graze the canopy as much as possible. This was needed especially at forest sites, when it was difficult to judge canopy height from the ground. After three minutes, we would fly the drone back, an assistant would block the entrance of the net with a large piece of cardboard, and the drone would be landed. The insects inside would then be collected inside ethanol filled tubes for future identification. At each site, time, temperature, humidity, weather, and wind speed were recorded.

### Lindgren funnel

In addition to the drone sampling, Lindgren funnels^20^ were placed at each site for six to seven days with generic antifreeze as an attractant and killing liquid. Adding these funnels to the study allow us to compare insect population abundance and composition from drone collections to a traditional entomology sampling technique. At wetland sites, the Lindgren funnel was placed at the same height and location that the drone would sample. At lake sites, the funnel was placed as close to the body of water as possible, usually attached to a tree on the border of the lake. At coniferous and deciduous forest sites, the funnels were placed in the understory—and not the canopy where the drone would sample.

### Statistical analyses

All analyses were performed used R Statistical Software (v4.1.2; R Core Team, 2021). The packages lme4^21^, lmerTest^22^, vegan^23^ and AICcmodavg^24^ were used. To analyse the insect yield in response to varying drone and net settings, we performed paired student’s t-tests on the data in order to determine which changes in settings most affected insect yield. These tests were done for each pair of settings; height (0 m vs. 1 m), speed (10 km/hr vs. 20 km/hr), net diameter (12 inches vs. 18 inches), and drone (Mavic 2 vs. Phantom IV). The t-tests were completed using log(x + 1) data in order to achieve normality and account for values of zero insect yield.

In order to analyse the effect of habitat and environmental factors on insect yield, eight generalized linear models using Poisson distribution were scored with an AICc (Supplementary Table S1). The four explanatory variables explored within the models were habitat, temperature, relative humidity, and time of day. Relative humidity (%) was transformed using an arcsine square root transformation to account for percentage, and time of day was changed into hours after midnight. Interactions were included in some models based on possible environmental explanations.

Finally, the Shannon-Weiner diversity index (H’), Simpson diversity index (D), Species richness (S), and Pielou’s evenness (J) were calculated for each habitat for both drone testing and Lindgren funnel output.

## Results

### Optimal drone settings

**Figure 2:**
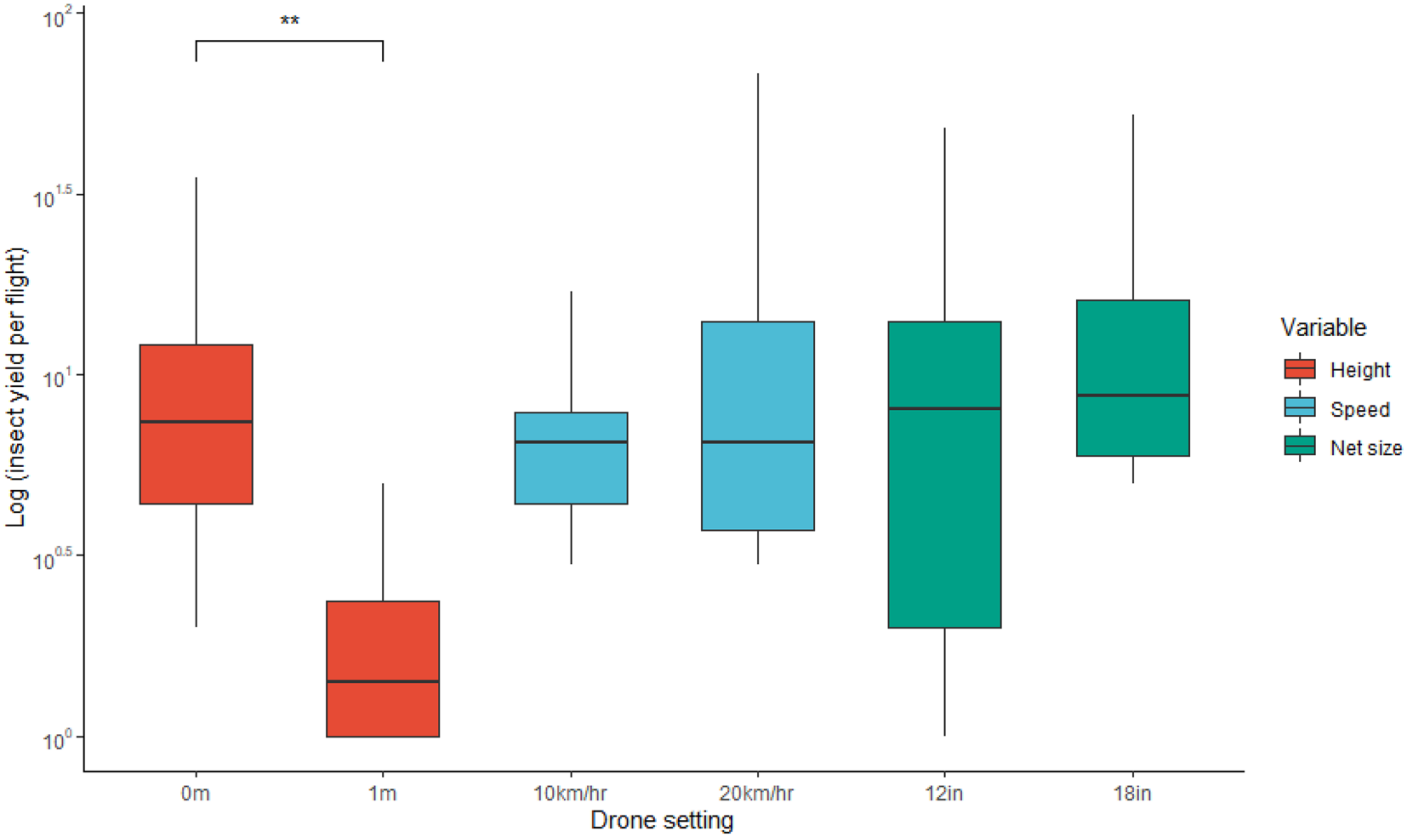
Boxplot of arthropod yields for different drone settings of height, speed, and net size (diameter). Boxes indicate median, first and third quartile, as well as minimum and maximum values. Significance stars are place based on paired t-tests of settings values, ** denotes *p* < 0.01 (only differences in height were significant with *p* = 0.0017).

During the September collection period to assess ideal drone settings, 167 individual insects in eight different orders were collected with two drones. Of the settings examined (height, speed, and net diameter), paired student’s t-tests reported only a significant difference between the insect yields of different heights (0 m vs. 1 m) (t_7_ = 4.9, *p* = 0.0017) (Fig. 2). There was also no significant difference found between the insect yield of different drones (Mavic 2 vs. Phantom IV) which was reflected in the model selection as well.

**Figure 3:**
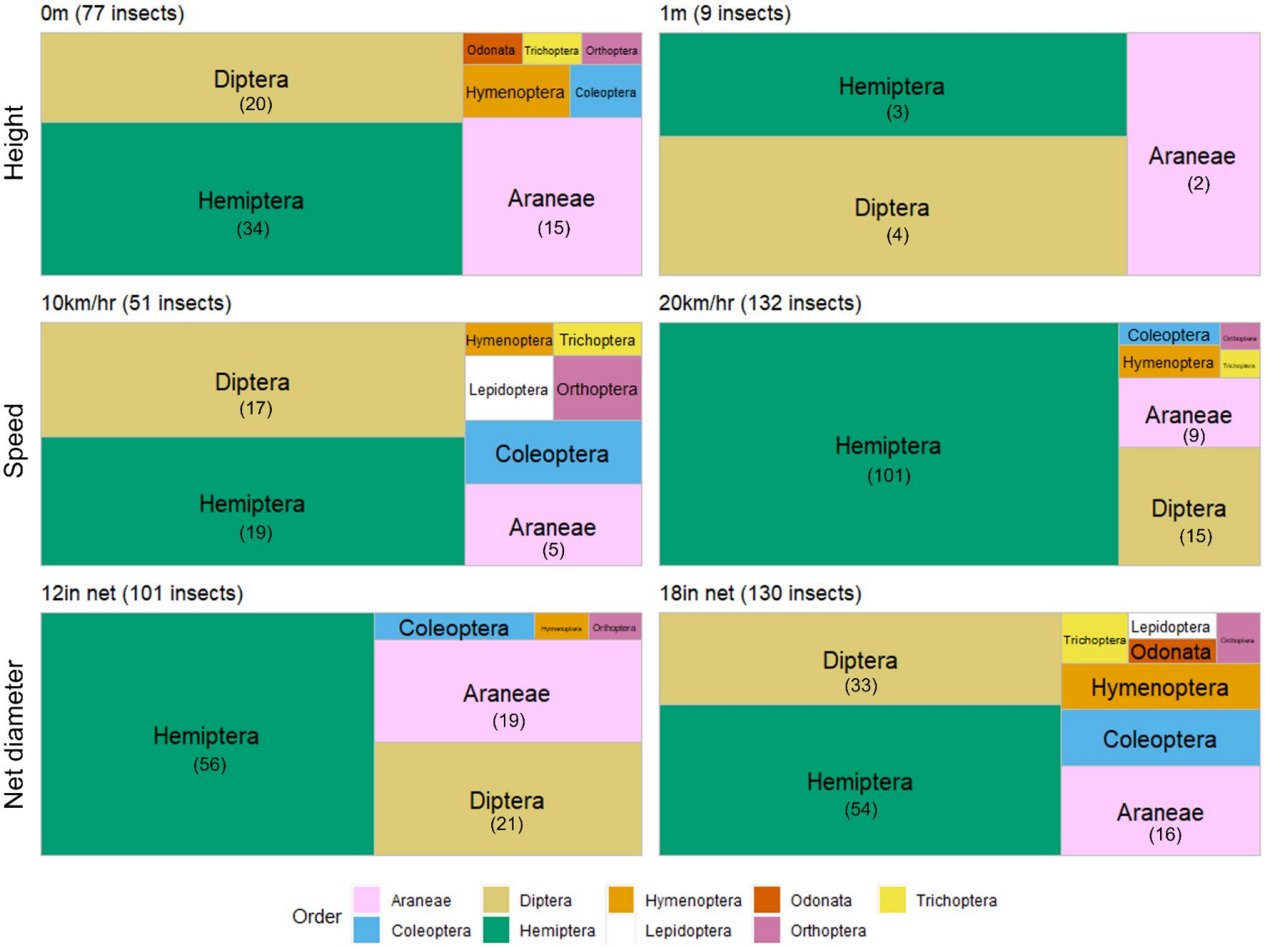
Treemap of insect sample composition collected by different drone settings; height (0 m vs. 1 m), speed (10 km/hr vs. 20 km/hr), and net diameter (12in vs. 18in). Default settings were 0 m, 10 km/hr, and 12 in. Boxes represent total proportion of the insect order compared to total insects caught using ta particular method. The total number of individuals collected of the top 3 orders collected by each setting are marked below the labels.

Major differences in diversity were not apparent between different drone settings of height, speed, and net diameter, though there were some notable minor differences (Fig. 3). Flying at one meter was the only setting that captured no insects of order Coleoptera or Hymenoptera. Due to the one-meter height setting catching so few insects (nine individuals over eight flights), the pattern represented in the tree map is unlikely to represent the true distribution of the habitat and is more so a reflection on the inefficiency of the setting. The diversity yield when flying at 20 km/hr showed the highest proportion of Hemiptera (77%, 101 insects) compared with other settings.

To determine if the second conical layer of tulle making up the drone net was effective at keeping the insects inside, insects in each layer were counted over a series of flights. Over 24 flights, 84% of the insect yield was found within the second layer of the net—the section that insects would be unable to easily fly out of.

### Insect abundance and diversity

**Figure 4:**
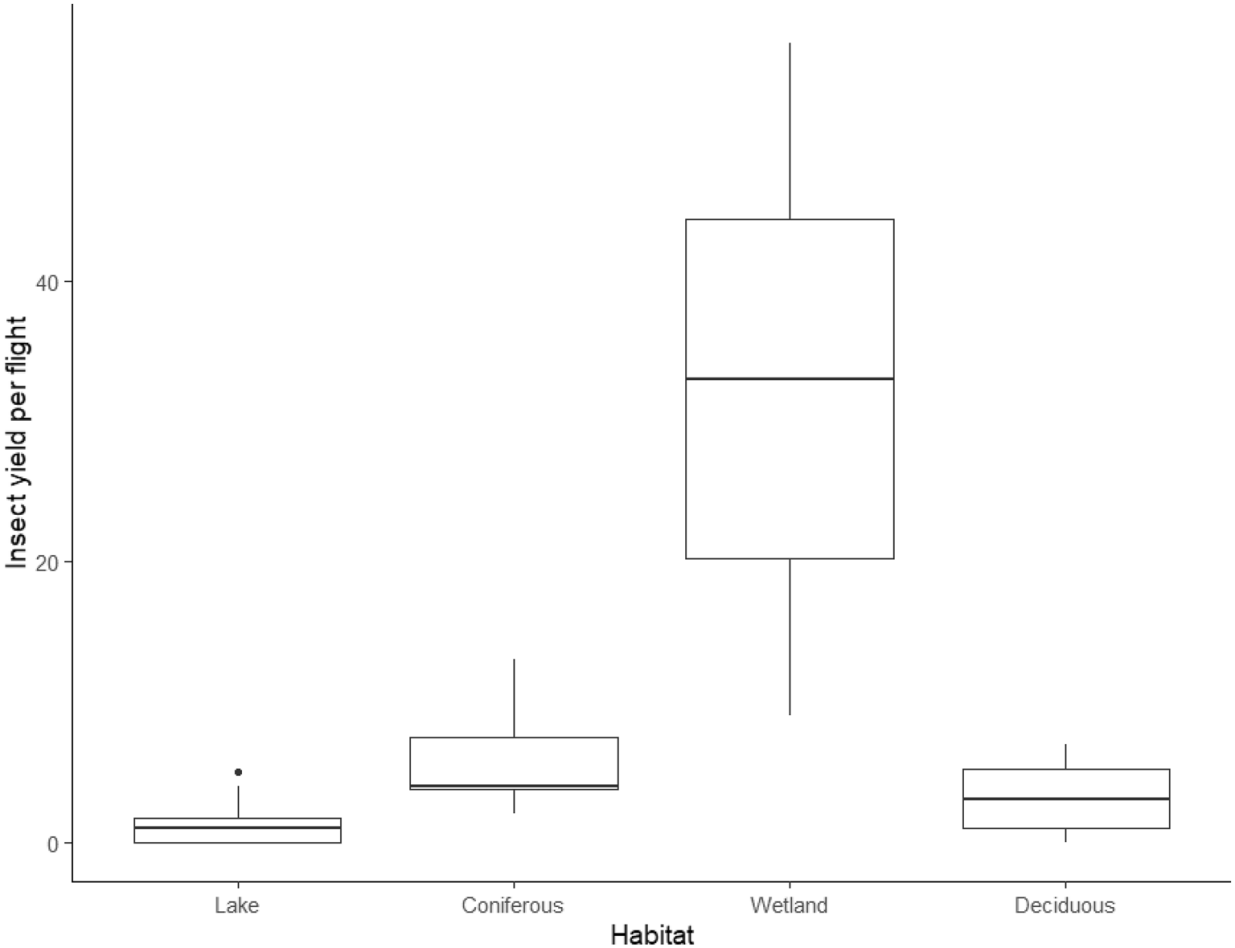
Boxplot of arthropod yield for the four study habitats collected after one 3-min drone flight with canopy contact. Boxes indicate median, first and third quartile, as well as minimum and maximum values.

In total, 516 individual insects representing 11 orders and 67 families were collected using drones during the June sampling period. On average, there were more insects collected per flight in the wetland habitats, followed by coniferous forests, deciduous forests, and finally lakes (Fig. 4). The difference between sites for average yield for each flight conducted was statistically significant with a one-way ANOVA test (F_2,44_ = 34.67, *p* < 0.001). An Akaike Information Criterion (AIC) model selection corrected for small sample size (AICc) scored the best model for insect yield as habitat type with an interaction with humidity, followed by habitat type and temperature (Supplementary Table S1).

**Figure 5:**
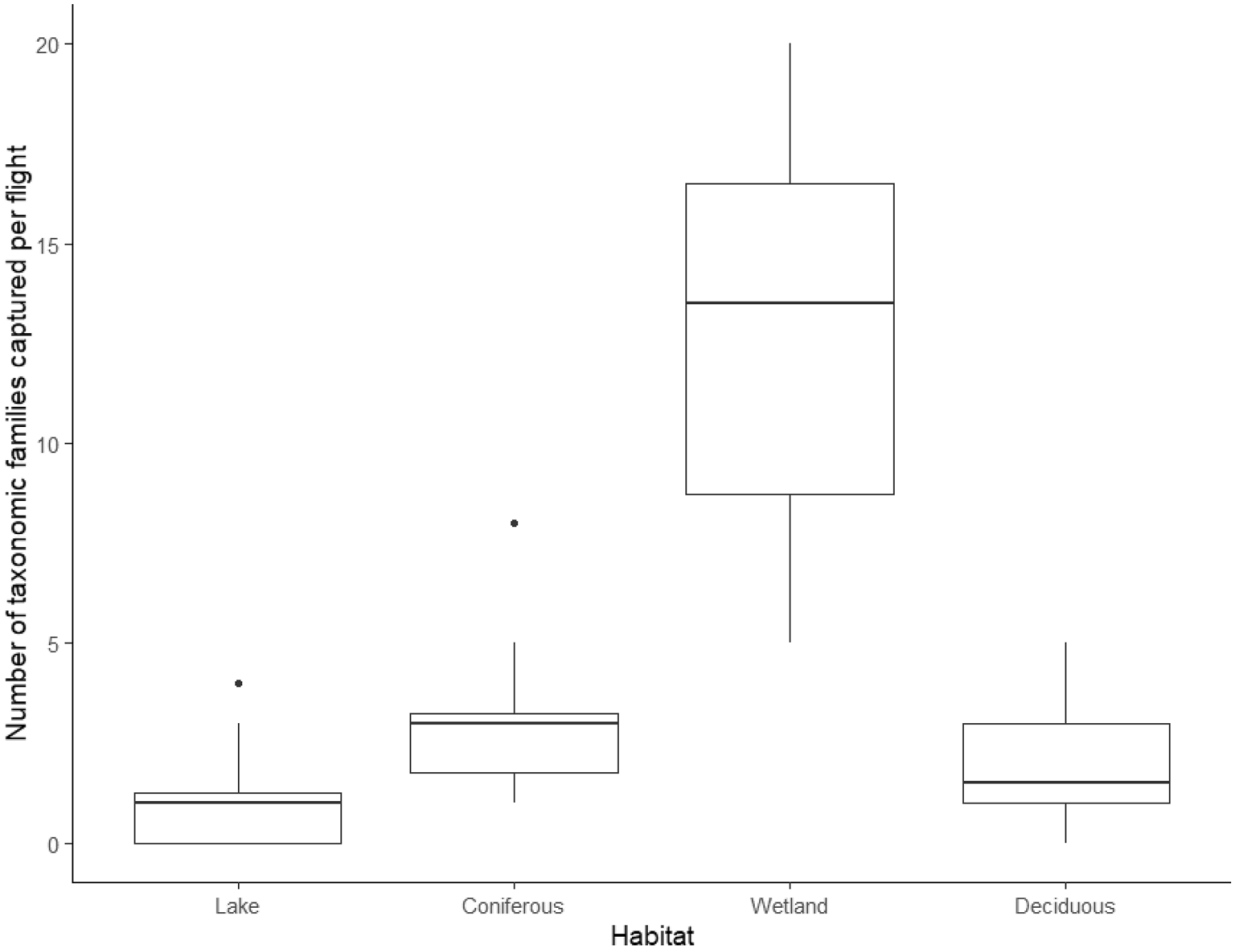
Boxplot of insect and arthropod families for the four study habitats collected after one 3-min drone flight with canopy contact. Boxes indicate median, first and third quartile, as well as minimum and maximum values.

The same pattern was found for diversity as in abundance, with more insect families collected per flight in the wetland habitats (60 total), followed by coniferous forests (21 total), deciduous forests (19 total), and lakes (10 total) (Fig. 5). This difference in diversity was found to be statistically significant with a one-way ANOVA test (F_2,44_ = 43.63. *p* < 0.001). However, family count alone without offsetting for abundance is not the best indicator of diversity. Both the Shannon-Weiner and Simpson diversity indices reported that wetlands had the highest diversity and species richness (H’ = 3.35, D = 0.940, S = 59), with lakes and coniferous forests reporting low diversity (Table 1).

**Table 1:** Diversity, richness, and evenness indices for the drone collection of the four habitats.

Habitat	Shannon-Weiner diversity index (H’)	Simpson diversity index (D)	Species richness (S)	Pielou’s evenness (J)
Coniferous forest	2.27	0.820	21	0.746
Deciduous forest	2.69	0.910	19	0.913
Wetland	3.35	0.940	59	0.823
Lake	2.23	0.886	10	0.969

**Figure 6:**
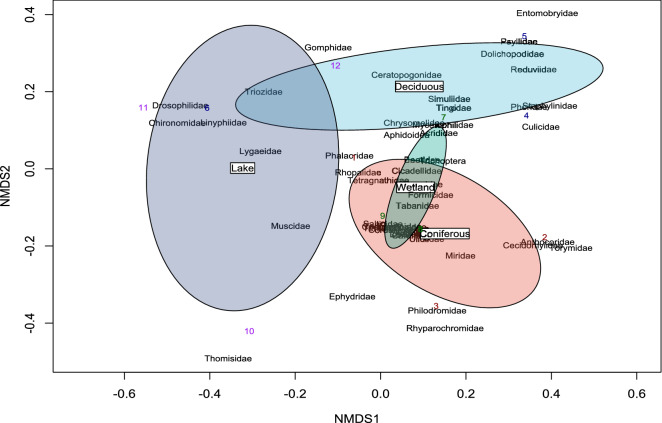
Non-metric multidimensional scaling (NMDS) of arthropod taxa observed in coniferous (red), deciduous (light blue), wetland (green), and lake (dark blue) habitats. Numbers represent sites within habitats (sites 1, 2, and 3 are coniferous, sites 4, 5, and 6 and deciduous, sites 7, 8, and 9 are wetlands, and sites 10, 11, and 12 are lakes). Points closer to each other are more similar in community composition than points farther apart.

Notably, the lake habitat had the highest evenness index (J), followed by deciduous forests, wetlands, and finally coniferous forests. Thus, while the lake sites have the lowest species richness, the diversity of families in the lake habitat is the most evenly distributed out of the four habitats. The composition of the different habitat communities was plotted with an NMDS plot (Fig. 6), showing that wetlands and coniferous forest sites have significant overlap between their insect family composition, with lake and deciduous sites also having a slight overlap.

### Lindgren funnel: comparison to drones

**Figure 7:**
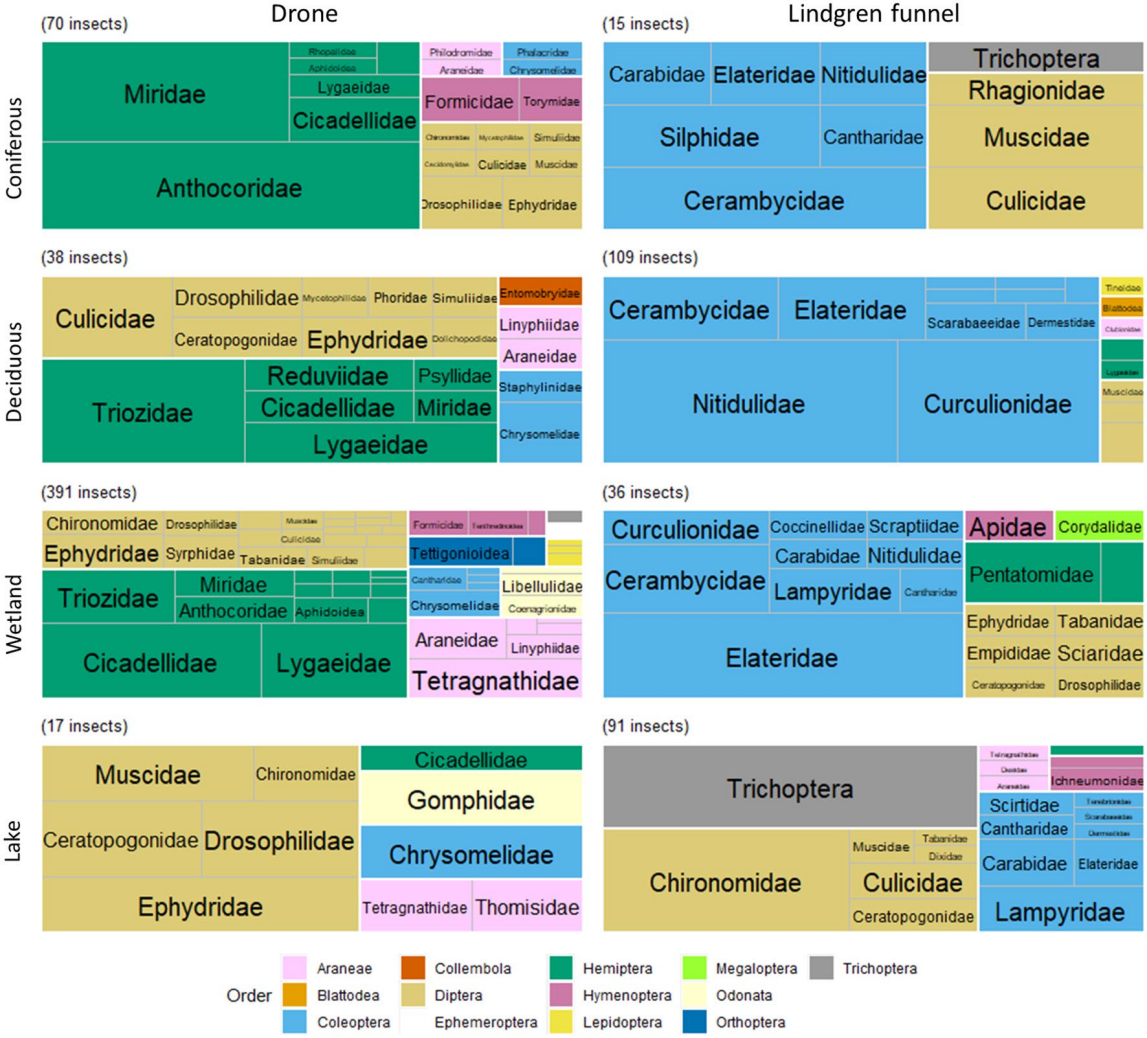
Treemap of arthropod sample composition collected by two methods; drone and hanging sweep net (left) and Lindgren funnel (right). Family and Order are grouped together for each habitat; Coniferous forest, deciduous forest, wetland, and lake. Boxes represent total proportion of the arthropod order compared to total arthropods caught at three different sites of each habitat. Total insects collected are marked above each treemap.

In total, 48 families in 9 orders were collected using Lindgren funnels during the June sampling period. The funnels collected 15 insects in 10 families at coniferous sites, 113 insects in 20 families at deciduous sites, 36 insects in 19 families at wetland sites, and 95 insects in 27 families at lake sites. The differences between the community composition caught by the drone and Lindgren funnels seem to remain constant throughout all 4 habitats (Fig. 7). Lindgren funnels had high proportions of beetles at all four habitats, compared to the proportions caught by drone. This was the case even in wetlands, where the funnel was placed at the same height as the drone flew and was expected to capture similar insects. Contrary to the samples caught by drones, the funnels reported highest species richness in lakes, and highest evenness in coniferous sites (Table 2). The wetland habitat remained the most diverse. The drone collection method had higher Shannon-Weiner (except at lakes) and Simpson (except at coniferous forests) diversity indices than the Lindgren funnel (Tables 1 and 2).

**Table 2:** Diversity, richness, and evenness indices for the Lindgren funnel collection of the four habitats.

Habitat	Shannon-Weiner diversity index (H’)	Simpson diversity index (D)	Species richness (S)	Pielou’s evenness (J)
Coniferous forest	2.21	0.880	10	0.960
Deciduous forest	1.99	0.787	19	0.674
Wetland	2.53	0.870	19	0.859
Lake	2.26	0.826	21	0.743

Regardless of the diversity of the samples caught, the drone collection method and Lindgren funnel method caught different numbers of insects at each habitat (Fig. 7). Drones collected disproportionately more insects at coniferous forests and wetlands compared to Lindgren funnels, while the funnels collected more insects at deciduous and lake sites. Since there was some discrepancy in the placement in the forest and lake habitats, the most equal comparison of methods would be at the wetland sites, where the Lindgren funnel was located in the same place and height that the drone sampled. The drone collection at wetlands yielded a total of 391 insects from the 3 sites, with each site averaging 130 insects per 12 combined minutes of flying. The Lindgren funnels at the wetland sites yielded a much smaller total of 36 insects, with each site averaging a yield of 12 insects per 7 days of the trap being set.

## Discussion

UAVs indeed proved to be a practical, efficient, and accurate tool in sampling insects within four different habitats in Quebec. Furthermore, different drone settings of speed, height, and net diameter may yield different insect orders, which can be useful in studies that aim to target specific insects. Nonetheless, only height, and not speed, net diameter or drone type influenced insect abundance. Compared with Lindgren funnels, drones were not only able to catch more insects in less time, but also a wider array of the insect community diversity.

Our study successfully shows the promise of using drones to collect forest and wetland canopy arthropods. More arthropods were collected flying at zero meters (grazing the canopy) than flying at one meter, while different speed, net size and drone type had less of an effect on insect yield (Fig. 2). The one-meter setting was expected to yield different arthropod diversity, such as fewer terrestrial families (ex. Araneae) and more aerial families (ex. Diptera) compared to the grazing zero-meter setting. However, the proportions of the top three orders (Diptera, Hemiptera, and Araneae) were similar among settings (Fig. 3). The capture of arachnids at one meter above the canopy can be explained by webs that are attached to taller foliage in proximity to the area, or spiders ‘ballooning’ in the airspace on silk threads^25^. Because canopy height was not always uniform, flying while grazing the canopy underneath the drone was at times lower than other parts of the canopy. Another explanation could be jumping spiders (ex. family Salticidae) which have been found to react to a disturbance or threat by leaping, possibly into the drone net^26^. Though the main three orders were in similar proportion, the one-meter setting caught five fewer orders in total than the zero-meter setting did. Flying at one meter was the only setting that captured no insects of order Coleoptera, Hymenoptera, or Orthoptera, suggesting that these orders spend time in and among the wetland canopy, and are seldom above the grassy canopy (Fig. 3). Most importantly, this setting only caught nine insects total over all flights, revealing itself to be an inefficient method of insect collection. This can be due to the number of insects available to be collected at each height. When flying at one meter, the net has access to only aerial insects in flight above the canopy (ex. flies). Flying while grazing the canopy, however, gives the researcher access to the same aerial insects in flight above the canopy, but also aerial insects in flight within the canopy (ex. bees), aerial insects at rest on the canopy (ex. leafhoppers), and terrestrial insects on the canopy (ex. ants). Thus, flying the drone while grazing the canopy opens the possibility of capturing three more insect groups compared to flying above the canopy. It is also possible that there are indeed many insects to be caught solely in the airspace, but that the ideal height for collecting insects strictly above the canopy is either less than or greater than one meter—which is the only height above the canopy that we tested.

This sampling period caught three total insects from order Odonata, with two of the three being caught with the 18-inch diameter net setting (Fig. 3). As these dragonflies are typically fast flyers and of large body size, perhaps the extra diameter of the larger net was helpful in increasing the chances of catching Odonates, though we do not have enough data to make solid conclusions. This would be a valuable line of future research for studies focused on dragonflies, or other large and fast-flying insects.

Flying the drone and hanging sweep net at 20 km/hr yielded the highest number and proportion of insects in the order Hemiptera, which are often found at rest within the canopy^27^. We speculate that the faster speed of the drone striking the grassy canopy more swiftly, thus giving the insects resting on the grasses less of an opportunity to evade the threat of the approaching net. Future studies targeting the collection of true bugs should utilize a faster drone speed in flight to optimize yield.

With 84% of insects found within the second layer of our net, we conclude that our novel net design with two layers of tulle is satisfactory in retaining insects and preventing most from escaping when landing the drone. In addition to the insects counted, we never witnessed any insects flying out during landing stages. We believe that our methodology of flying the drone in quickly and covering the opening of the net with cardboard before landing the drone, in addition to the extra layer of netting, was successful at retaining the insects caught. Determining how to fly the drone and net over the two forest canopy habitats was a challenge. When flying, it was impossible for the drone camera to look both forward—to see obstacles coming up, and downwards—to see how close the net was hanging regarding the top of the canopy. For this reason, we used a second drone as a spotter for the first, the pilot of which could give instructions on moving up or down. Forest canopies were particularly difficult, as the height from one tree to the next was always different, the drone had to be constantly adjusted. We experienced many snags on branches, although they were not damaging to the net or drone. Once we became comfortable flying the drone low enough to graze the canopy, snagging became a common occurrence that was easily remedied. In fact, snagging the net probably helped in the collection of insects on those branches—a technique that could be honed and used in future studies using nets and drones over forest canopies.

Over our 12 days of sampling habitat canopies with drones, we were able to determine that wetlands had the highest diversity and abundance of the four habitats examined, with lake habitats showing the lowest Shannon-Weiner Diversity index (H’), and the highest Pielou’s evenness index (J). It is unsurprising that lakes showed the most even distribution of families, as is often the case with habitats having low species richness, as there are less competitors that could dominate the habitat^28^. Habitat, humidity, and temperature were the most important variables affecting drone insect yield, with habitat being the common variable in all high scoring models. Wetlands had by the far the most insects collected, in addition to the highest diversity and species richness. This can be explained simply by the plant composition in wetlands compared to the other habitats. While coniferous and deciduous forests are dominated by a few species (and lakes have little to no vegetation over the water) wetlands can host a wide variety of plant species. Because insect diversity correlates with plant richness and abundance, wetlands can provide shelter and sustenance for many more groups of insects that the other habitats we studied^29^.

Lindgren funnels disproportionately collected insects from order Coleoptera (Fig. 7). Although Lindgren funnels have been used in papers reporting results focused on insects of orders Hemiptera^30–33^ and Diptera^34–36^, it is unclear whether some were targeted studies or all simply bycatch of the funnel from other experiments. Instead, Lindgren funnels are overwhelmingly used in Coleoptera studies as the funnels resemble a tree and attracts various wood-boring beetles^37–41^. This attraction explains the large number and proportion of beetles caught in funnels in this study. However, diversity indices show that in three of four habitats, drones collect a higher diversity sample than the Lindgren funnels (Tables 1 and 2). Thus, though Lindgren funnels are undoubtedly effective at collecting beetles from the environment, our results indicate that the drone collection method is preferable when seeking an accurate representation of the insect diversity of the habitat. Studies focused on Coleoptera could also employ this method, which would be helpful in determining the status and proportion of beetles within the population and compared to other insect orders.

In addition to the larger diversity collected by drones, the temporal advantage of this technique over the funnels can not be understated. During our study, it took three Lindgren funnel traps established for seven days to collect a total of 36 insects at the wetland sites (0.001 insect collected per minute). Comparatively, at the same height and placement, drones were able to collect 391 insects in only a combined 36 min (10.9 insects collected per minute) (Fig. 7). This large difference in both yield and time scale demonstrates that the drone collection method is vastly more efficient at arthropod sampling compared to the Lindgren funnels.

While this study was successful at validating the usefulness of drones in canopy entomology studies and insect collection in general, it does have its limitations. Optimal drone settings were only examined at wetland grassy canopy sites, and it is possible that the drone might perform differently within different habitats. For example, grazing the canopy at 20 km/hr might result in high insect yield at wetlands, where the lack of obstacles made it relatively easy to fly quickly. But the same settings may be unrealistic and prone to net snagging when sampling over other habitats, such as the coniferous forest canopy. Furthermore, Lindgren funnels were an acceptable comparison to drone collection for yield and diversity at some habitats, however it was impossible to get the funnels up into the canopy where sampling took place at coniferous and deciduous sites. There is no doubt that the advantage of this method lies in its accessibility, speed, and safety—studies that need more precise and fine sampling might not benefit from drones.

Overall, our research demonstrates that drones are an efficient and accurate tool in collecting a wide diversity of insects above the canopies of different habitats. Benefits included rapidly and safely sampling the airspace while drawbacks included battery life limiting the duration of sampling. If this new technique is integrated into the field of entomology, canopy studies can be done much more often, for less money, and more safely than they have been done using other techniques. In 2019, a review of the potential causes of decline of aerial insectivores concluded that insect declines and changes in high quality prey availability could be a large driver of insectivore declines^9^. However, there is a lack of research detailing insect trends over time. The drone collection method used in this study could provide the missing link between the need for more research of aerial canopy insects and the limitations of the current methodology in entomology. This technique can be used in conjunction with aerial insectivore surveys and diet studies to begin to determine the relationship between declining predators and prey. Future research may also use and add to our guidelines to customize drone and net settings for studies targeting specific insect orders or families.

## Supplementary Information

Supplementary Information.

## Data Availability

The datasets generated during and analysed during the current study are available from the corresponding author on reasonable request.
