# Supplementary material for: Optimal settings and advantages of drones as a tool for canopy arthropod collection
Source: Sci Rep. 2022 Oct 26;12:18008. doi: 10.1038/s41598-022-22446-z (PMC9606009; doi:10.1038/s41598-022-22446-z)
Supplement: Supplementary file 1 — Supplementary Information. [file 41598_2022_22446_MOESM1_ESM.docx]

**Supplementary material**

**Supplementary Table S1**: AICc table for results of all models explaining the yield of arthropods collected by UAVs at different habitats. Models were built based on the relevant biological explanations, “+” symbols denote the addition of variables in the model, “*” symbols denote interactions between variables. Complex models with multiple interactions and variables were unable to be explored due to the sample size. Bolded models show a ∆AICc of less than 2.

| Model | K | AICc | ∆AICc | AICcWt | Cum.Wt | LL |
| --- | --- | --- | --- | --- | --- | --- |
| **Habitat*Humidity** (see code below) | 8 | 251.36 | 0.00 | 0.88 | 0.88 | -115.01 |
| Habitat + Temperature | 5 | 256.70 | 5.33 | 0.06 | 0.95 | -122.35 |
| Habitat + Temperature + Humidity | 6 | 257.61 | 6.25 | 0.04 | 0.98 | -121.36 |
| Habitat+Humidity | 5 | 260.74 | 9.38 | 0.01 | 0.99 | -124.37 |
| Habitat*Temperature | 8 | 261.02 | 9.66 | 0.01 | 1.00 | -119.84 |
| Habitat | 4 | 339.37 | 88.01 | 0.00 | 1.00 | -165.22 |
| Null | 1 | 952.31 | 700.94 | 0.00 | 1.00 | -475.11 |

glm(yield ~ hab*humidity, family = poisson(link = "log"), data=data)
